# Feasibility study of advanced focused cardiac measurements within the emergency department

**DOI:** 10.1186/s13089-018-0093-4

**Published:** 2018-05-25

**Authors:** Joe Betcher, Al Majkrzak, Jim Cranford, Ross Kessler, Nik Theyyunni, Rob Huang

**Affiliations:** 1 Department of Emergency Medicine, Lake Michigan Emergency Specialists, Mercy Health Muskegon, Muskegon, USA; 2 Department of Emergency Medicine, Emergency Physicians Medical Group, St. Joseph Mercy Ann Arbor, Ann Arbor, USA; 3 Department of Psychiatry, University of Michigan, Ann Arbor, MI USA; 4 Department of Emergency Medicine, University of Michigan, Ann Arbor, MI USA

**Keywords:** Ultrasound, Echocardiogram, VTI, Diastolic, Focused cardiac ultrasound, Point of care ultrasound

## Abstract

**Background:**

This study aims to compare the increased time needed to perform advanced focused cardiac measurements in the emergency department, including diastolic heart failure evaluation via E/E′, and cardiac output with LVOT/VTI. Patients with pertinent cardiopulmonary symptoms in the emergency department had a focused cardiac ultrasound performed by the emergency department ultrasound team. The ability to obtain basic cardiac windows, evaluate for effusion, systolic ejection fraction, and right-sided heart pressures were recorded. Advanced measurements, along with time to obtain all images and the training level of the provider, were recorded.

**Results:**

Fifty-three patients were enrolled. Basic focused cardiac windows were able to be obtained in 80% of patients. The average 4-window focused cardiac ultrasound took 4 min and 49 s to perform. Diastolic measurements were able to be obtained in 51% of patients, taking an average of 3 min and 17 s. Cardiac output measurements were able to be obtained in 53% of patients, taking an average of 3 min and 8 s.

**Conclusion:**

The ability to obtain these images improved with increasing level of training. Performing both cardiac output and diastolic measurements increased the time with bedside ultrasound by 6 min and 25 s, and were able to be obtained in slightly over half of all ED patients.

## Background

Focused cardiac ultrasound (FOCUS) has become increasingly utilized by emergency medicine (EM) physicians to evaluate undifferentiated patients within the emergency department (ED). The ability to accurately diagnose patients presenting with acute dyspnea or hypotension has been shown to be aided by use of FOCUS [1–3]. Recent studies have shown that EM physicians are able to interpret complex FOCUS findings with high levels of accuracy [4]. Furthermore, the ability of EM physicians to perform and interpret cardiac findings such as ejection fraction, right heart strain/function, diastolic function, fluid status, valvular dysfunction, and aortic dissection has been studied and proven in the literature [5–12].

**Fig. 1:**
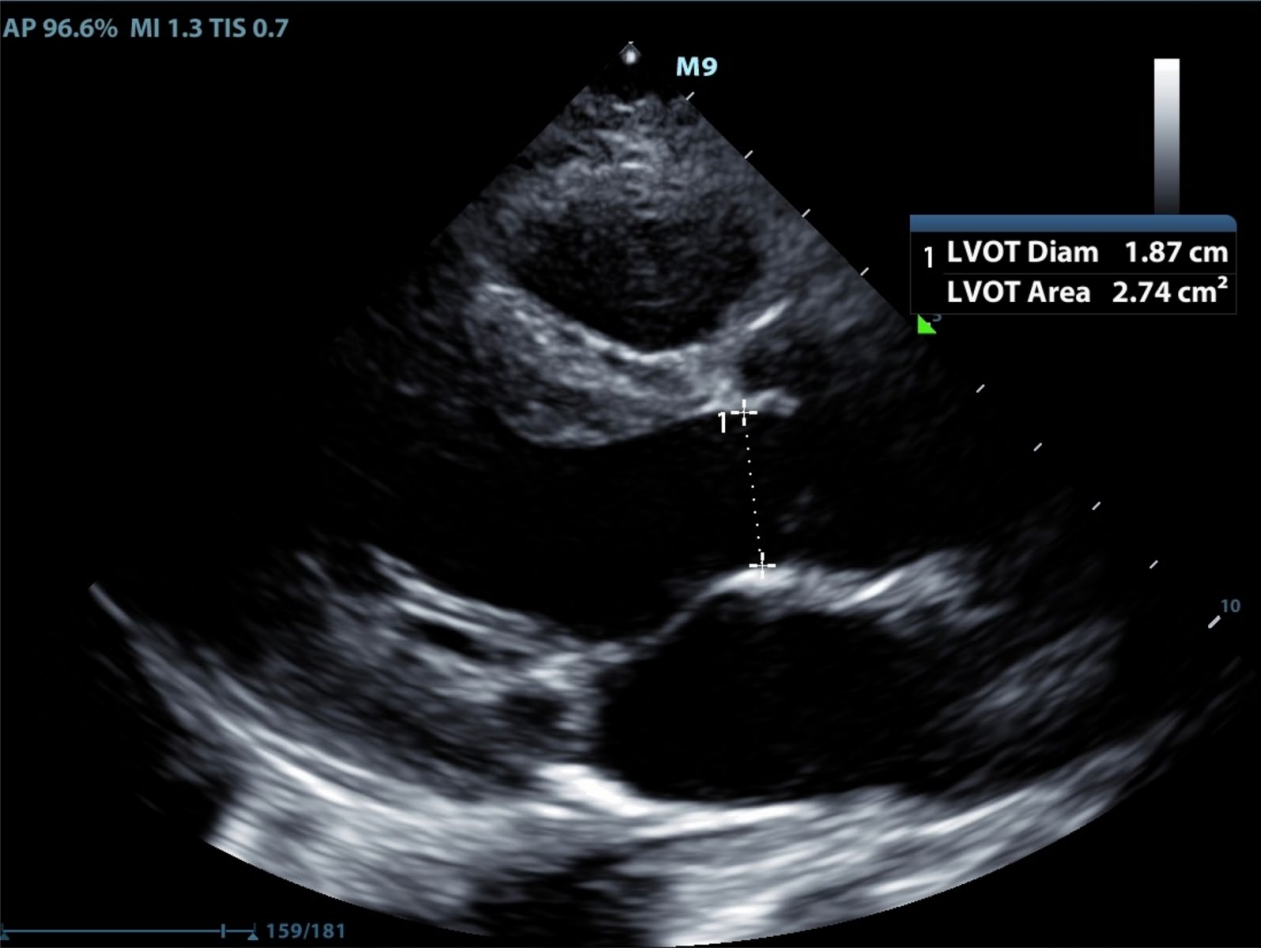
Measurement of LVOT in PSLV

**Fig. 2:**
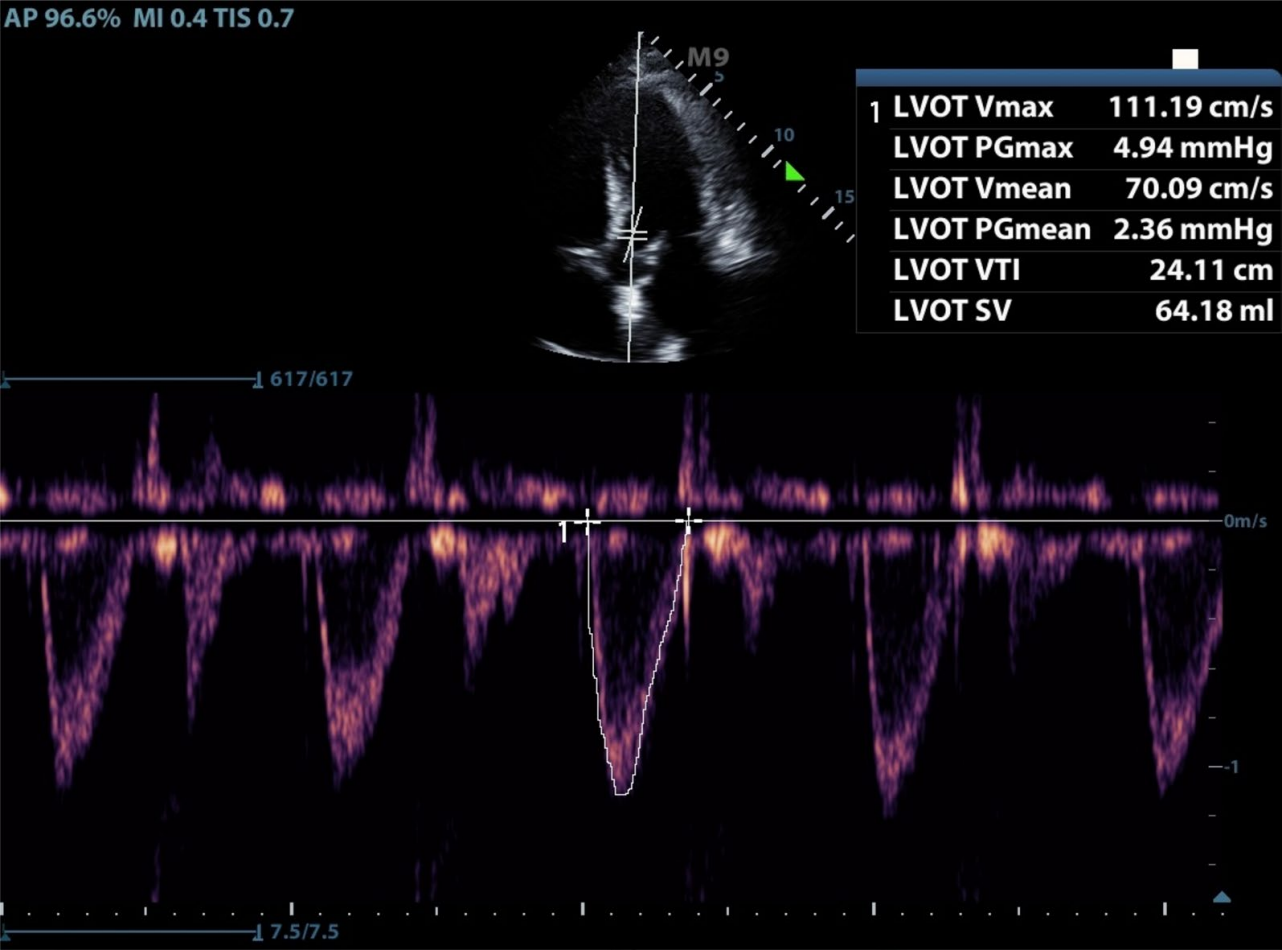
Measurement of VTI in apical view

Fluid status evaluation is a recommended indication for FOCUS [13]. Although there are multiple methods to evaluate fluid status in the cardiopulmonary patient, including history and physical exam, FOCUS for inferior vena cava measurement, central venous pressure measurement, stroke volume variation, and passive leg raise, there remains no clear foolproof method for this evaluation. Cardiac velocity time integral (VTI) is used to calculate stroke volume in the evaluation of volume responsiveness, and can be measured through basic cardiac windows [14] (Figs. 1, 2). Prior research has shown that EM physicians can perform cardiac VTI measurements accurately [15].

**Fig. 3:**
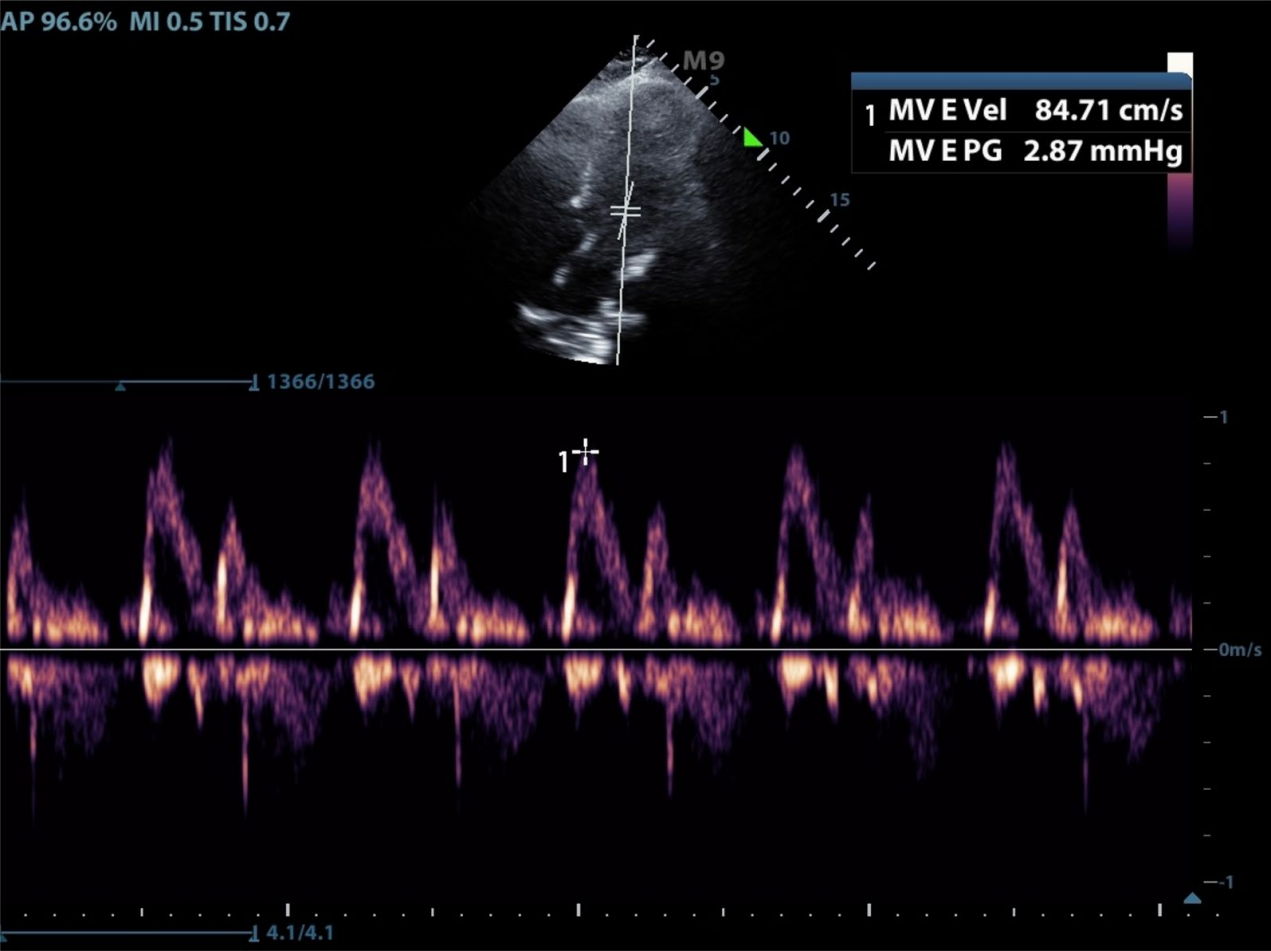
Measurement of mitral valve E in apical view

**Fig. 4:**
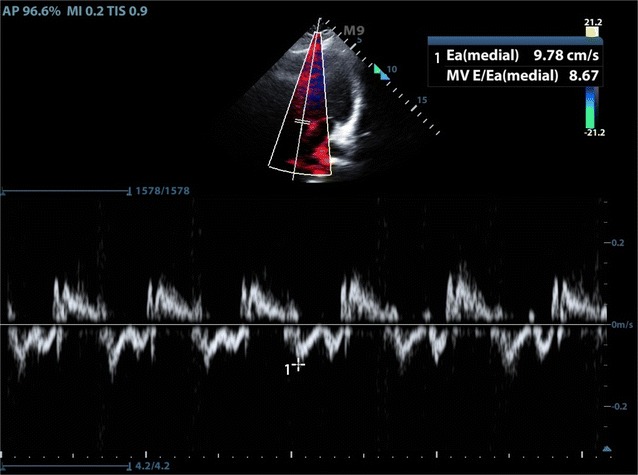
Measurement of mitral valve E' in apical view

Similarly, the work-up of diastolic heart failure within the ED can be elusive, as the systolic ejection fraction seen on basic windows can appear normal. Approximately 50% of congestive heart failure (CHF) exacerbations are due to diastolic failure, leading to frequent presentations within the ED. EM physicians have also been shown to be able to perform and interpret diastolic CHF measurements with high sensitivity [8] (Figs. 3, 4). Tasked with providing care to many patients simultaneously, FOCUS must be performed efficiently in order to add value in the diagnostic work-up of the patient with cardiopulmonary complaints. Each additional FOCUS measurement adds to the time needed to evaluate each patient. With the recent evidence that EM physicians can perform and interpret complex FOCUS measurements, we sought to prove the feasibility of performing these measurements within the ED. We evaluated both the frequency and speed that physicians at varying skill levels were able to obtain measurements to calculate both VTI and diastolic heart failure. As multiple prior studies have proven that FOCUS impacts decision making, this study only sought to prove feasibility of performance of advanced cardiac measurements, not the impact they have on decision making [16–18].

## Methods

This prospective, observational study was conducted in an academic emergency department with a residency training program. The institutional review board approved this study (HUM00113390, 2016). Participation in this study was voluntary and no compensation was offered. Patients were verbally consented prior to inclusion in the study. Patient data were gathered over a 2-month time period. All sonographic images were obtained by Mindray^®^ M9 ultrasound machines (Mindray Ltd., Shenzhen, China) available to the emergency department for regular patient care use. A Mindray phased array (P4-1c) probe was used for obtaining all images.

Patients were selected via a sample of patients who presented to the ED with a chief complaint of chest pain or dyspnea. Patients older than 18 years of age were eligible for the study. When the initial ED provider deemed that FOCUS was necessary in the ED for further aid with narrowing the differential diagnosis, the EM-based ultrasound team was consulted. This team consisted of an ultrasound fellow, along with an EM resident. This team was available for 8-h blocks in the ED, from the hours of 12:00 P.M. to 8 P.M., on weekdays. This availability was based on the point of care ultrasound rotation for EM resident education.

Basic demographic information, including the patient’s age, weight, and BMI, was gathered. This team then performed FOCUS, and attempted to obtain parasternal long (PSLA), parasternal short (PSSA), apical five-chamber, and subxiphoid views. A gross visual interpretation of the systolic ejection fraction, right ventricular systolic heart strain pertaining to visual interpretation of abnormal ventricular septal movement in the PSSA and apical views, and visual assessment of pericardial effusion was made by the performing resident/fellow. Following this, left ventricular outflow tract (LVOT) diameter in a PSLA, and the VTI in the apical view were obtained, for calculation of stroke volume. Finally, the E and E′ values were obtained in the apical five-chamber view, for measurement of possible diastolic heart failure. The interpretations and measurements were recorded, and reported back to the EM provider. Given that patients were evaluated in real time within the emergency department, while also undergoing further work-up with laboratory studies and other possible imaging studies, only either the resident or fellow performed the ultrasound on the individual patient.

These measurements were chosen as they represent advanced cardiac measurements that require advanced training in FOCUS, yet can potentially aid EM physicians in obtaining complex diagnoses. The time needed to perform LVOT, VTI, E, and E′ was the primary outcome of this study.

Prior to performing the study, residents were given a 20-min didactics on performance of LVOT/VTI and diastolic measurements. They were then allowed to practice on a standardized model prior to performance of any FOCUS on patients with cardiopulmonary complaints. The residents were given hands-on instruction from the fellow on obtaining the advanced cardiac measurements on the standardized model. All residents, varying from Post Graduate Year (PGY) 1–4, had varying amounts of experience with both FOCUS and point of care ultrasound. All residents involved in this study were rotating through a month-long point of care ultrasound rotation as part of their EM residency training. The images and measurements on a patient made by residents were independent from the fellow on the team. Similarly, the ultrasounds performed by the fellows did not have interpretations by the resident.

The cardiac window images and LVOT/VTI and E/E′ images were then over-read by multiple ultrasound-fellowship trained EM physicians, to determine if images were of suitable quality, as this is the primary outcome of the study. The interpretation and image acquisition had to be complete to be considered adequate. Recorded images were deemed adequate/inadequate based on image gain, depth, appropriate LVOT diameter measurement, appropriate doppler placement for VTI, E, and E′ images, and appropriate doppler waveforms for VTI, E, and E′ images. All criteria for LVOT/VTI/E/E′ needed to be deemed adequate to be considered of appropriate quality. The studies deemed of appropriate quality for use with clinical decision making with regard to LVOT/VTI and E/E′ measurements, by an ultrasound expert were included in the data analysis. These ultrasound-fellowship trained physicians currently practice at the institute performing the study. They did not collect individual data on any patients or assist with any collection of data. The collected data from the residents/fellows was then analyzed by a statistician using a Test of Between-Subjects Effect to evaluate for significance.

## Results

A total of 53 patients were enrolled in the study. 19 of these had an ultrasound performed by a junior resident (PGY 1–2), 17 had an ultrasound performed by a senior resident (PGY 3–4), and 17 had an ultrasound performed by an ultrasound fellow. Eight PGY 1–2 residents performed ultrasounds with a mean of 2.4 ultrasounds per resident. Seven PGY 3–4 residents performed ultrasounds with a mean of 2.4 ultrasounds per resident. Two fellows performed ultrasounds with a mean of 8.5 ultrasounds per fellow. The age, weight, and BMI of the patients were similar in characteristics among the 3 provider groups (Table 1).

**Table 1:** Average patient characteristics

	Age (years)	Weight (kg)	BMI (kg/m^2^)
Junior resident	55.9	96.5	31.9
Senior resident	59.3	86.0	30.5
Fellow	50.3	84.0	30.1

The level of training was compared with regard to time to obtain 4 windows via a Test of Between-Subjects Effect, and the level of training was found to be statistically significant, *F* (2,47) = 17.4, *p* < .05 (Tables 2 and 3).

**Table 2:** Basic cardiac window obtained by level of training

	% PSLV	% PSSV	% apical	% subxiphoid
Junior resident	68	63	63	79
Senior resident	88	88	88	71
Fellow	88	92	82	82

**Table 3:** Time in seconds to obtain cardiac measurements by level of training

	Basic 4 view (s)	LVOT/VTI (s)	E/E′ (s)	Complete echo (s)
Junior resident	400	219	249	792
Senior resident	282	249	217	677
Fellow	193	144	122	446

Following blinded review via emergency medicine ultrasound faculty, junior residents were able to obtain LVOT/VTI/E/E′ measurements in 26% of patients. 79% of the images regarding LVOT/VTI/E/E′ measurements by junior residents were deemed of adequate quality by ultrasound faculty. Junior residents took on average 219 s to perform LVOT/VTI measurements, 249 s to perform E/E′ measurements, and 792 s for the entire FOCUS.

Senior residents were able to obtain LVOT/VTI/E/E′ measurements in 53% of patients. 76% of the images regarding LVOT/VTI/E/E′ measurements by junior residents were deemed of adequate quality by ultrasound faculty. Senior residents took on average 249 s to perform LVOT/VTI measurements, 217 s to perform E/E′ measurements, and 677 s for the entire FOCUS.

Fellows were able to obtain LVOT/VTI/E/E′ measurements in 65% of patients. 89% of images regarding LVOT/VTI/E/E′ by fellows were deemed of adequate quality by ultrasound faculty. Fellows took on average 144 s to perform LVOT/VTI measurements, 122 s to perform E/E′ measurements, and 446 s for the entire FOCUS (Table 4).

**Table 4:** Advanced cardiac measurements obtained by level of training

	LVOT (%)	VTI (%)	Both LVOT/VTI (%)	E (%)	E′ (%)	Both E/E′ (%)
Junior resident	42	37	37	37	37	37
Senior resident	65	53	53	59	59	59
Fellow	82	65	65	65	65	65

The level of training was compared with regard to time to obtain LVOT/VTI via a Test of Between-Subjects Effect, and the level of training was found to be statistically significant, *F* (2,22) = 4.6, *p* < .05. The level of training was also compared with regard to time to obtain E/E′ via a Test of Between-Subjects Effect, and the level of training was found to be statistically significant, *F* (2,25) = 5.6, *p* < .05. Finally, the level of training was also compared with regard to time to obtain all images and measurements via a Test of Between-Subjects Effect, and the level of training was found to be statistically significant, *F* (2,23) = 7.6, *p* < .05.

## Discussion

In this study, we evaluated the ability to obtain advanced FOCUS measurements including LVOT/VTI and E′/E′, and the time required to obtain them by an ED physician. The percentage of successful measurements of LVOT/VTI and E/E′ was lower than initially expected. In the previous study by Dinh et al., ED patients that had valvular disease, were unable to lie supine, or were unable to lie in the left lateral decubitus position were excluded, which potentially explains their ability to obtain adequate VTI measurements in 90% of patients [15]. The average patient BMI for all groups in our study was > 30, placing them into the obese category, leading to an increased challenge with obtaining images [19]. Many patients were in respiratory distress, and often on non-invasive or mechanical ventilation. They were frequently unable to assist with any positioning to obtain improved cardiac windows. Performance of the ultrasound was done early in the patient’s work-up for the treating ED physician, leaving little time for resuscitation prior to obtaining cardiac windows and measurements. We believe this provides external validity to this study, having patients that were often in critical condition and with an undifferentiated cardiopulmonary diagnosis, which is the rationale for the urgently performed FOCUS within the ED.

To answer the question of ‘How fast is fast enough’ for obtaining advanced cardiac measurements, this largely depends on the resources available to the EM physician utilizing FOCUS. Acuity level of patients, patients seen per hour, ICU/cardiology consultation availability, and inpatient bed availability are only several of the factors that are taken into account when managing a department and performing a patient’s work-up, and subsequently planning for their disposition. Given the widely varying resources within each ED, each provider needs to weigh the pros/cons of performing FOCUS with regard to their efficiency of their work-flow. As the fellows were able to obtain these LVOT/VTI and E/E′ measurements accurately in approximately 2 min in greater than 50% of patients, it is reasonable to perform these when appropriate, based on the provider’s resources. While an ultrasound fellow may have more FOCUS experience than most EM physicians, they were still relatively inexperienced in performing these measurements, and we expect that accuracy and time to gather the images would continue to improve, regardless of the provider’s prior training. Junior residents were able to obtain the LVOT/VTI and E/E′ measurements in approximately 4 min. In light of diagnostic uncertainty in patients with a complex cardiac history in their ED presentations, these 4 min may be beneficial in starting the correct treatment with regard to their fluid status.

Although frequently discussed in recent Free Open Access Medical Education (FOAM) discussions, these measurements are rarely obtained in the clinical setting within the emergency department [20, 21]. The physicians obtaining these studies were in a teaching setting, and were not responsible for other patient care during the time of FOCUS. Further research is needed to determine if ED physicians are able to perform these measurements while on clinical shifts and responsible for other patient care, and if so, will influence decision making for the critically ill patient.

Our results also indicate a statistically significant increase in success rates with ability to gather adequate measurements as physicians progressed through their training, and also a statistically significant decrease in time to gather those images. While not unexpected that as physicians advance in their training that their skill levels will improve, this helps to demonstrate that ED physicians can improve their ability to perform advanced measurements in FOCUS dramatically with increased training.

Limitations for our project include lack of blinding to the EM physician performing the ultrasound. As our physicians knew they were being evaluated, they may have been more motivated to obtain accurate images. While a previous study by Unluer et al. demonstrated that ED physicians could diagnose diastolic failure with good sensitivity, our study was also largely focused on the time taken to perform these studies, rather than diagnostic accuracy, as our goal was to determine if these measurements are feasible in a busy ED [8]. A more detailed study may track the changes in time needed to perform these by individual physicians as they progress in their skill level with regard to performing advanced cardiac measurements. An additional limitation is with blinded review of the recorded measurements by expert reviewers. Reviewers were asked to approve only images/measurements that were of quality suitable for clinical decision making. This exact level of quality is difficult to quantitate, and will likely vary among expert reviewers.

## Conclusion

The scope of FOCUS for EM physicians continues to expand, with patients presenting with increasingly complex cardiopulmonary disease. Their cardiac function and fluid status often remains elusive throughout the beginning of their ED work-up. These advanced cardiac measurements take training to competently perform, and could add additional time to the patient’s evaluation with FOCUS, but can be performed in an efficient manner once learned, in the appropriate clinical setting. These measurements performed by ED physicians may become a feasible tool for the critically ill cardiopulmonary patient.
